# 25th Anniversary of the Americans with Disabilities Act — July 2015

**Published:** 2015-07-31

July 2015 marks the 25th anniversary of the passage of the Americans with Disabilities Act (ADA), signed into law on July 26, 1990, by President George H.W. Bush. ADA prohibits discrimination against persons with disabilities in all areas of their everyday lives, such as work, school, transportation, communication, recreation, and access to state and local government services. When first enacted, ADA defined a disability as a “physical or mental impairment that substantially limits one or more of the major life activities.”(1)

During the last 2 decades, multiple national surveys measured disability in various ways because of substantial differences in the conceptualization and definition of disability. More recently, several national health surveys incorporated a recommended standard set of questions assessing functional types of disability.

In recognition of ADA’s milestone anniversary, this issue of MMWR includes a report using the first data available on functional types of disability in a state-based health survey. It includes prevalence of functional disability using a standard set of disability questions rather than measuring disability in a nonspecific manner. This report presents the percentage of adults with any disability and with specific types of disabilities by state and key demographic characteristics (e.g., sex, age, race/ethnicity).

For more information on disability research and surveillance and state and national disability programs and resources, access the CDC’s Disability and Health Branch, available at http://www.cdc.gov/ncbddd/disabilityandhealth/.

## References

[b1-777] 1Americans with Disabilities Act of 1990, Pub. L. 101–336, 104 Stat. 328 (July 26, 1990) [amended January 1, 2009]. Available at http://www.ada.gov/pubs/adastatute08.htm

